# STEP Levels Are Unchanged in Pre-Frontal Cortex and Associative Striatum in Post-Mortem Human Brain Samples from Subjects with Schizophrenia, Bipolar Disorder and Major Depressive Disorder

**DOI:** 10.1371/journal.pone.0121744

**Published:** 2015-03-18

**Authors:** Thomas A. Lanz, J. Julie Joshi, Veronica Reinhart, Kjell Johnson, Lonnie E. Grantham II, Dmitri Volfson

**Affiliations:** 1 Pfizer Research & Development, Cambridge, MA, United States of America; 2 Arbor Analytics, Ann Arbor, MI, United States of America; UTHSCSH, UNITED STATES

## Abstract

Increased protein levels of striatal-enriched tyrosine phosphatase (STEP) have recently been reported in postmortem schizophrenic cortex. The present study sought to replicate this finding in a separate cohort of postmortem samples and to extend observations to striatum, including subjects with bipolar disorder and major depressive disorder in the analysis. No statistically significant changes between disease and control subjects were found in STEP mRNA or protein levels in dorsolateral prefrontal cortex or associative striatum. Although samples were matched for several covariates, postmortem interval correlated negatively with STEP protein levels, emphasizing the importance of including these analyses in postmortem studies.

## Introduction

Striatal-enriched tyrosine phosphatase (STEP) is a brain-specific, developmentally regulated tyrosine phosphatase. Two functional splice variants exist; STEP61 is a membrane-bound isoform found throughout the forebrain, and STEP46 is a cytosolic isoform found only in striatum [1]. The substrates of STEP include the GluR2 subunit of the AMPA [2] and the NR2B subunit of the NMDA receptor [3], and thus position STEP as a downstream negative regulator of glutamate receptor signaling. Transcriptional profiling of STEP KO mice has provided additional support for the role of STEP in the NMDA signaling pathway [4]. A recent report showed increased STEP protein levels assessed by Western blot in postmortem schizophrenic cortex [5]. Given the NMDA hypofunction hypothesis of schizophrenia [6, 7], increased STEP activity would be hypothesized to reduce functional NMDA signaling. Hence inhibition of this enzyme could represent a therapeutically attractive approach to schizophrenia treatment, particularly if levels are abnormally elevated in the disease state.

The present study aimed to replicate the postmortem human results in a separate postmortem cohort of schizophrenic brains, and include matched subjects with bipolar disorder and major depressive disorder (MDD). Dorsolateral prefrontal cortex (PFC) and associative striatum (dorsal caudate) were evaluated for disease state STEP mRNA and protein level changes.

## Methods

Detailed methods are provided in Supplementary Material. All procedures were approved by the University of Pittsburgh Committee for the Oversight of Research and Clinical Trials Involving the Dead and the Institutional Review Board for Biomedical Research. PFC and associative striatum were obtained from the University of Pittsburgh brain bank, curated by Dr. David Lewis (http://www.tnp.pitt.edu/pages/donationpg_mb.htm). All procedures for donation and processing of tissue and for evaluation and diagnosis have been previously described [8]. Multiple sections were cut from 19 tetrads of subjects with schizophrenia, bipolar disorder, MDD matched with controls according to age, gender and PMI (postmortem interval). Subject demographic information is presented in Table 1. Total RNA was prepared in a trizol-chloroform extraction from one set of sections, while protein was prepared in another set according to published methods [9]. Briefly, the samples were homogenized by sonication in lysis buffer (125 mM Tris HCl pH 7, 2% SDS, 10% glycerol plus a protease/phosphatase inhibitor cocktail from Thermo). The samples were then incubated at 70°C for 10 min followed by a 10 min centrifugation at 16,000 rcf. Protein concentration was determined by DC Lowry assay (BioRad Life Science Research, Hercules, CA). Protein samples (10 μg per lane) were boiled in Novex Tris-Glycine SDS Sample Buffer (2x) for 5 min before being resolved by SDS PAGE (26-well, 4–20% gradient Tris-Glycine gel) in Tris-Glycine SDS running buffer. Each protein sample was run on 3 gels, n = 3 per gel based pilot experiments to assess inter- and intra-gel variability. Gels were run at 150 V for 90 min before being transferred to a nitrocellulose membrane (0.2 μm) at 180 V for 70 min in NuPage 20X Transfer buffer, and probed by Western blot for STEP (custom polyclonal antibody directed against the sequence “KEYDIPGLVRKNRYKT” within the PTP domain) and cyclophilin B (Thermo Fisher), a mitochondrial protein loading control. Secondary antibodies with different wavelengths were used for STEP (680 nm) and cyclophilin B (800 nm). Blots were scanned on an Odyssey infrared scanner (Li-Cor, Lincoln, NB). Local background was subtracted from all bands, and STEP bands were normalized to cyclophilin B.

**Table 1:** Human subject demographics and sample metadata. Values are mean ± standard deviation (n = 19 per group): RIN = RNA Integrity Number; Medication at time of death (Meds ATOD): C = centrally acting medications (Benzodiazepines, anticonvulsants, antidepressants, antipsychotics, lithium), O = other medications, U = unknown, N = none; MOD = Manner of Death (N = natural; A = accidental; S = suicide).

	Control	Bipolar	Major Depressive Disorder	Schizophrenia
Gender	10 M, 9 F	10 M, 9 F	10 M, 9 F	10 M, 9 F
Race	18 W, 1 B	19 W, 0 B	18 W, 1 B	13 W, 6 B
Age (years)	48.1 ± 10.6	46.3 ± 9.5	45.2 ± 10.1	45.1 ± 8.5
PMI (hours)	19.5 ± 5.1	21.3 ± 6.6	20.1 ± 6.0	20.1 ± 6.9
Brain pH	6.6 ± 0.2	6.6 ± 0.2	6.6 ± 0.2	6.4 ± 0.4
RIN	7.6 ± 0.67	7.4 ± 0.73	7.4 ± 0.79	7.0 ± 0.83
Tobacco ATOD	5 Y, 14 N	9 Y, 5 N, 5 U	7 Y, 12 N	12 Y, 7 N
Meds ATOD	7 O, 12 N	15 C, 2 O, 2 N	10 C, 8 N, 1 U	16 C, 1 O, 2 N
MOD	15 N, 4 A	6 N, 5 A, 8 S	9 N, 3 A, 7 S	8 N, 4 A, 7 S

RT-PCR was performed using Taqman assays Hs00377290_m1, Hs00377913_m1 and Hs00986488_g1 (STEP), (Life Technologies) and relative quantities determined using standard curves. Parvalbumin was assayed with Taqman assay Hs00161045_m1 (Life Technologies). The geometric mean of two endogenous controls was used to normalize expression values: Hs00161446_m1 (RPN1) and Hs03043885_g1 (RPL13A; Life Technologies). These were selected as the most stable endogenous controls using NormFinder software after analyzing a panel of potential endogenous control genes in all samples.

Protein and RNA data were analyzed by one-way analysis of covariance (ANCOVA) mixed model, which incorporated case (disease state with 4 levels: control, schizophrenia, bipolar, and major depressive disorder) as a main factor, and PMI, age, and gender as covariates, as well as interactions between the main factor and covariates. The response variables were log base 10 transformed to correct model residuals, which were found to be non-normally distributed as assessed by residual plots. Non-significant covariates were removed sequentially from the model, and planned multiple comparisons tests were used to assess differences between disease states. All statistical tests were performed two-tailed at a 5% level of significance.

## Results

**Fig 1:**
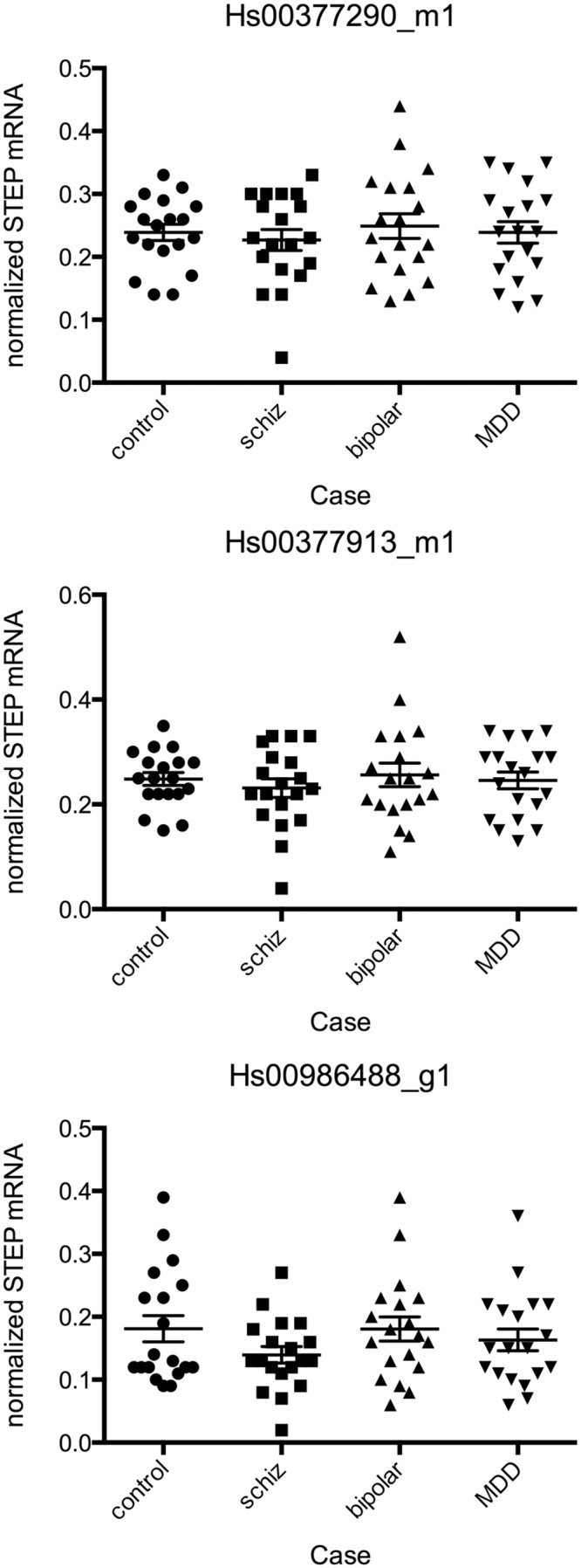
STEP61 mRNA levels were measured by RT-PCR in PFC from subjects with schizophrenia, bipolar disorder, MDD, or age-matched controls using probe sets specific for different sequences within the STEP transcript (titles are Taqman assay numbers). No statistically significant changes were observed using any of the probe sets for STEP.

RT-PCR was performed in PFC using assays targeting three different sites throughout the STEP61 transcript, but no statistically significant differences were identified (Fig. 1). None of the covariates had a systematic effect upon STEP61 mRNA levels (Table A in S1 File), though pH had a significant effect upon 1 of the 3 assays (F = 4.043, p = 0.0487). As these samples had been used in a microarray analysis previously (GEO Dataset GSE53987), the probe sets for STEP were examined in both PFC and STR (these probe sets will detect both STEP61 and STEP46). No statistically significant differences were observed in either region for any of the STEP-specific probe sets on the Affymetrix chip; RMA-normalized values for the probe set with the highest intensity are shown in Fig. A in S1 File. As a positive control for the present collection of brains from subjects with schizophrenia, parvalbumin mRNA was measured by RT-PCR (G). As has been demonstrated in multiple other postmortem schizophrenic cohorts [10–14], a significant disease-associated reduction in parvalbumin mRNA was measured in pre-frontal cortex (Fig. B in S1 File, F = 16.25, p<0.0001), with significant differences from control measured in schizophrenia (p<0.001) and bipolar disorder (p = 0.0037).

**Fig 2:**
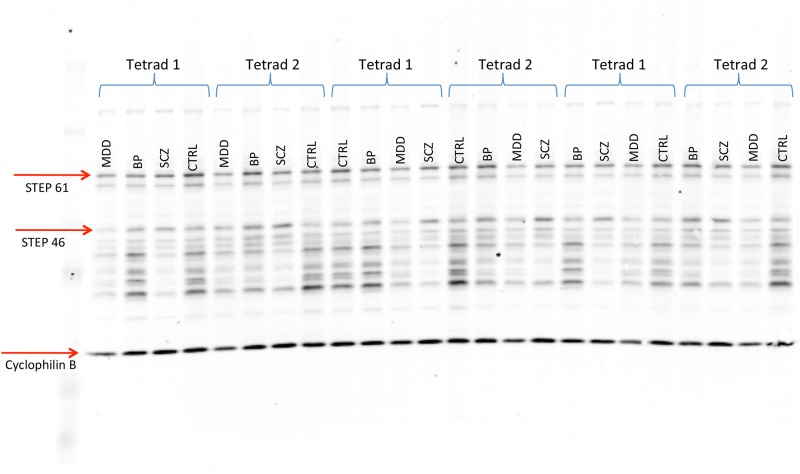
Example western blot from striatal samples.

**Fig 3:**
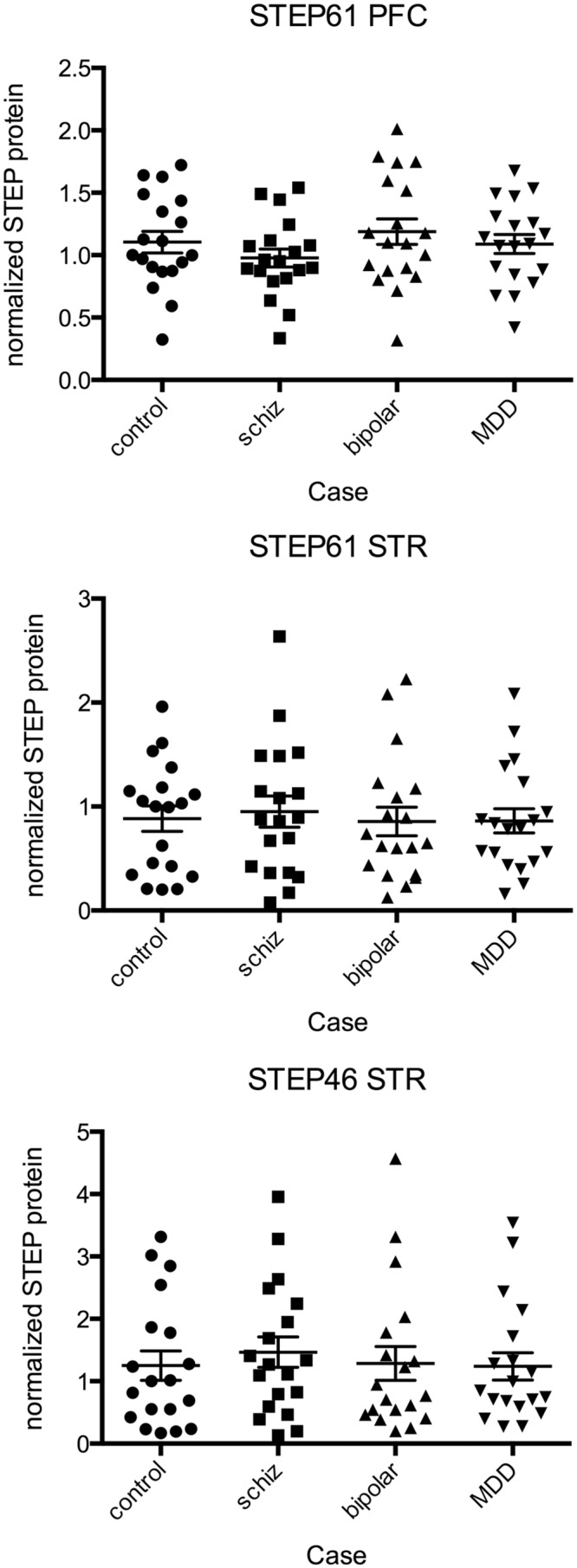
Quantitation of STEP protein levels in human postmortem brain tissue from subjects with schizophrenia, bipolar disorder and major depressive disorder. STEP61 was quantified in Brodmann Area 46 (A) and associative striatum (B), and STEP46 was quantified only in associative striatum (C), as it was not present in the cortex. STEP levels were normalized to a cyclophilin loading control. Intensity values were scaled to the median of the control group. Each point on the graph represents a single subject (each subject was run on 3 separate gels, with 3 replicates per gel).

**Fig 4:**
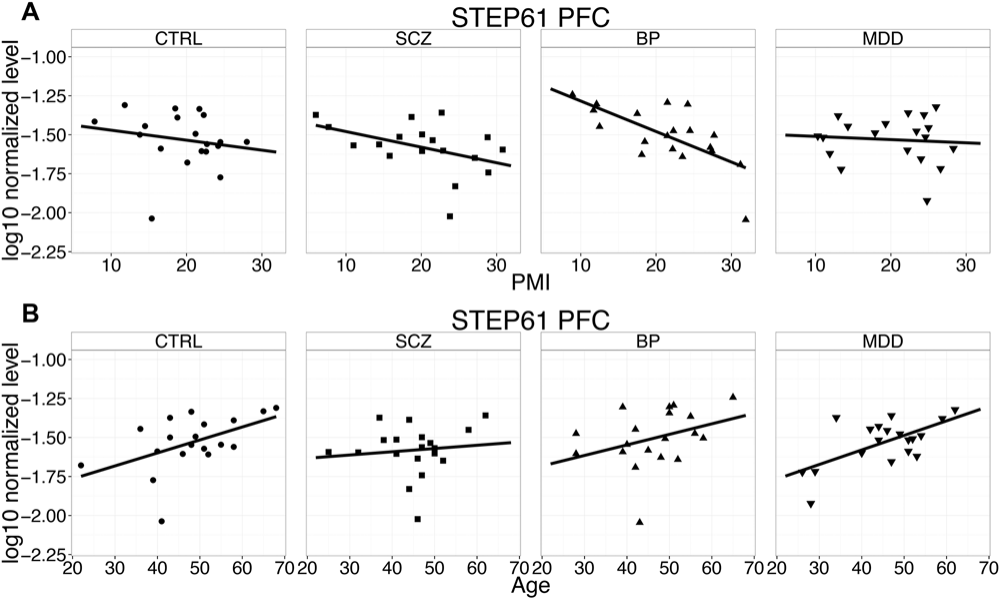
Postmortem samples showed dependence between postmortem interval PMI (A) and Age (B) and STEP protein levels analyzed by Western blot in PFC from bipolar (BP), control (CTRL), major depression (MDD) and schizophrenic (SCZ) subjects.

STEP protein levels were measured by western blot in PFC and STR, with each sample representing 3 times per gel across 3 gels in a randomized order, retaining matched tetrad groupings within the same gel (example blot shown in Fig. 2). Bands were selected based on molecular weight, and were validated in a separate blot using STEP knockout mouse brain tissue and recombinant human STEP46 and STEP61 (Fig. C in S1 File). For STEP61 protein in PFC (Fig. 3), the full ANCOVA model incorporating interaction between covariates and CASE factor did not show any statistically significant interaction effects (Table B in S1 File), therefore interaction terms were excluded. The simplified model revealed a strong association between STEP61 levels and two covariates, PMI (F = 10.77, p = 0.0011) and age (F = 11.24, p = 0.0009) (Fig. 4) and relatively weaker association with gender (F = 4.52, p = 0.0341). The CASE factor was not significant, (F = 1.09, p = 0.3611). Planned multiple comparisons tests for pairwise differences among covariate-adjusted means did not reveal any significant findings (Table C in S1 File). Similarly, for STEP61 probe in the striatum the full model did not show interaction between covariates and CASE (Table D in S1 File). The simplified model again revealed PMI was a strong covariate (F = 20.04, p<0.0001). In contrast with cortex, gender was significant (F = 6.89, p = 0.0089) while age was not was not (F = 0.17, p = 0.6822). The CASE factor was not significant, (F = 0.06, p = 0.9790). Planned multiple comparisons tests for pairwise differences among covariate-adjusted means did not reveal any significant findings (Table E in S1 File). Finally, results for STEP46 probe in striatum were quantitatively similar (Tables F, G in S1 File).

## Discussion

NMDA hypofunction has been suggested to play a key role in schizophrenia neurophysiology, with potential therapeutic implications for agents that can normalize NMDA signaling [7, 15–17]. As a downstream regulator of signaling in this pathway, STEP thus represents an interesting potential target with relevance to schizophrenia. A previously published report showed a significant increase in STEP protein levels in two separate cohorts of postmortem brains from subjects with schizophrenia [5]. The present data, however, failed to replicate this finding in a separate cohort of subjects. Furthermore, no statistically significant changes were observed in striatum from subjects with schizophrenia. Bipolar disorder and MDD also had no change in STEP level relative to well-matched control subjects. Transcript levels were similarly unchanged between disease states and control in PFC and striatum. Parvalbumin mRNA levels were significantly reduced in PFC from these samples, however, as has been observed in PFC from several other cohorts of postmortem schizophrenia samples [10–14].

The previous analyses of STEP protein levels were performed in PFC from a cohort whose mean age was 70 years old and whom were mostly non-medicated at the time of death, and the mean PMI was approximately 12 hours (n = 14) [5]. Most of the schizophrenic subjects in the present study were medicated, the mean age was 45–48, and mean PMI was approximately 20 hours (n = 19). Thus age, PMI and medication status could represent potential variables that would influence STEP levels. The second cohort in the Carty et al. publication was of similar age, PMI and medication status to the present dataset, but the region examined was anterior cingulate cortex (n = 12). As most post-mortem analyses of changes in schizophrenia are focused on PFC, it is unknown to what extent changes in STEP or other proteins observed in PFC occur in anterior cingulate cortex either due to the disease itself or the medication history of the subject.

While the samples for the present study were matched according to a number of covariates, analysis of these covariates independent of matching revealed a negative correlation between postmortem interval and STEP protein levels. Increased protein degradation over time is not uncommon, and great care must be taken to ensure that case and control cohorts are evenly matched. If the distribution is uneven between case and control, the regression of such a covariate could end up producing an artifact.

The present work sought to understand whether significant alterations in the regulation of STEP mRNA or protein could be observed in postmortem brains of subjects with psychiatric disease. Microarray and RT-PCR probe sets targeting multiple regions of the STEP transcript yielded no significant differences in mRNA, and Western blot analysis showed no significant changes in protein in PFC or striatum in schizophrenia, bipolar disorder, or MDD. Thus while a prior manuscript has reported an increase in STEP protein in schizophrenia, the effect was not robust enough to be reproduced in this independent cohort. These results do not preclude the possibility that enzyme activity could be altered in the disease state, but further investigation would be necessary to determine whether such a change is occurring in schizophrenia or another psychiatric disease.

## Supporting Information

S1 File Fig. A. Normalized expression data for the Affymetrix U133_Plus2 probeset with the highest intensity for STEP are shown for PFC (top) and associative striatum (bottom). No groups had a statistically significant difference from the control group in any probeset. **Fig. B**. Parvalbumin mRNA is significantly reduced in PFC from schizophrenia subjects as measured by RT-PCR (**p<0.01, ***p<0.0001). **Fig. C**. Top: Western blot using STEP KO mouse striatal lysates to validate the polyclonal STEP antibody used to measure human STEP protein. Bottom: Western blot probing with the same STEP antibody evaluating recombinant human STEP46, full-length STEP61, and a variant of STEP61 missing a 23 amino acid proline-rich juxtamembrane domain (PRJD). **Table A**. STEP mRNA levels in cortex: results for testing significance of fixed effects in mixed model. **Table B**. STEP61 protein in cortex: initial results for testing significance of fixed effects in the full mixed model, including all interactions between covariates and the CASE factor. **Table C**. STEP61 protein in cortex: results of multiple comparisons tests for pairwise differences amongst covariate-adjusted means. **Table D**. STEP61 protein in striatum: initial results for testing significance of fixed effects in the full mixed model, including all interactions between covariates and the CASE factor. **Table E**. STEP61 protein in striatum: results of multiple comparisons tests for pairwise differences amongst covariate-adjusted means. **Table F**. STEP46 protein in striatum: initial results for testing significance of fixed effects in the full mixed model, including all interactions between covariates and CASE factor. **Table G**. STEP46 protein in striatum: results of multiple comparisons tests for pairwise differences amongst covariate-adjusted means.
(DOCX)

## References

[1] Boulanger LM, Lombroso PJ, Raghunathan A, During MJ, Wahle P, Naegele JR. Cellular and molecular characterization of a brain-enriched protein tyrosine phosphatase. The Journal of neuroscience: the official journal of the Society for Neuroscience. 1995 2;15(2):1532–44. Epub 1995/02/01. eng. 7869116 10.1523/JNEUROSCI.15-02-01532.1995 PMC6577844

[2] Zhang Y, Venkitaramani DV, Gladding CM, Kurup P, Molnar E, Collingridge GL, et al The tyrosine phosphatase STEP mediates AMPA receptor endocytosis after metabotropic glutamate receptor stimulation. The Journal of neuroscience: the official journal of the Society for Neuroscience. 2008 10 15;28(42):10561–6. Epub 2008/10/17. eng. 10.1523/JNEUROSCI.2666-08.2008 18923032 PMC2586105

[3] Braithwaite SP, Adkisson M, Leung J, Nava A, Masterson B, Urfer R, et al Regulation of NMDA receptor trafficking and function by striatal-enriched tyrosine phosphatase (STEP). The European journal of neuroscience. 2006 6;23(11):2847–56. Epub 2006/07/06. eng. 16819973 10.1111/j.1460-9568.2006.04837.x

[4] Reinhart VL, Nguyen T, Gerwien R Jr, Kuhn M, Yates PD, Lanz TA. Downstream effects of striatal-enriched protein tyrosine phosphatase reduction on RNA expression in vivo and in vitro. Neuroscience. 2014 10 10;278:62–9. 10.1016/j.neuroscience.2014.08.002 25130559

[5] Carty NC, Xu J, Kurup P, Brouillette J, Goebel-Goody SM, Austin DR, et al The tyrosine phosphatase STEP: implications in schizophrenia and the molecular mechanism underlying antipsychotic medications. Translational psychiatry. 2012;2:e137 10.1038/tp.2012.63 22781170 PMC3410627

[6] Gonzalez-Burgos G, Fish KN, Lewis DA. GABA neuron alterations, cortical circuit dysfunction and cognitive deficits in schizophrenia. Neural Plast. 2011;2011:723184 10.1155/2011/723184 21904685 PMC3167184

[7] Lin CH, Lane HY, Tsai GE. Glutamate signaling in the pathophysiology and therapy of schizophrenia. Pharmacology, biochemistry, and behavior. 2012 2;100(4):665–77. 10.1016/j.pbb.2011.03.023 21463651

[8] Glausier JR, Kimoto S, Fish KN, Lewis DA. Lower Glutamic Acid Decarboxylase 65-kDa Isoform Messenger RNA and Protein Levels in the Prefrontal Cortex in Schizoaffective Disorder but Not Schizophrenia. Biological psychiatry. 2014 5 29. 10.1016/j.biopsych.2014.05.010 PMC4247819 24993056

[9] Curley AA, Arion D, Volk DW, Asafu-Adjei JK, Sampson AR, Fish KN, et al Cortical deficits of glutamic acid decarboxylase 67 expression in schizophrenia: clinical, protein, and cell type-specific features. The American journal of psychiatry. 2011 9;168(9):921–9. 10.1176/appi.ajp.2011.11010052 21632647 PMC3273780

[10] Fung SJ, Webster MJ, Sivagnanasundaram S, Duncan C, Elashoff M, Weickert CS. Expression of interneuron markers in the dorsolateral prefrontal cortex of the developing human and in schizophrenia. The American journal of psychiatry. 2010 12;167(12):1479–88. 10.1176/appi.ajp.2010.09060784 21041246

[11] Hashimoto T, Bazmi HH, Mirnics K, Wu Q, Sampson AR, Lewis DA. Conserved regional patterns of GABA-related transcript expression in the neocortex of subjects with schizophrenia. The American journal of psychiatry. 2008 4;165(4):479–89. 10.1176/appi.ajp.2007.07081223 18281411 PMC2894608

[12] Iwamoto K, Bundo M, Kato T. Altered expression of mitochondria-related genes in postmortem brains of patients with bipolar disorder or schizophrenia, as revealed by large-scale DNA microarray analysis. Hum Mol Genet. 2005 1 15;14(2):241–53. Epub 2004/11/26. eng. 15563509 10.1093/hmg/ddi022

[13] Mellios N, Huang HS, Baker SP, Galdzicka M, Ginns E, Akbarian S. Molecular determinants of dysregulated GABAergic gene expression in the prefrontal cortex of subjects with schizophrenia. Biological psychiatry. 2009 6 15;65(12):1006–14. 10.1016/j.biopsych.2008.11.019 19121517

[14] Sibille E, Morris HM, Kota RS, Lewis DA. GABA-related transcripts in the dorsolateral prefrontal cortex in mood disorders. Int J Neuropsychopharmacol. 2011 7;14(6):721–34. 10.1017/S1461145710001616 21226980 PMC3388740

[15] Gilmour G, Dix S, Fellini L, Gastambide F, Plath N, Steckler T, et al NMDA receptors, cognition and schizophrenia—testing the validity of the NMDA receptor hypofunction hypothesis. Neuropharmacology. 2012 3;62(3):1401–12. 10.1016/j.neuropharm.2011.03.015 21420987

[16] Gonzalez-Burgos G, Lewis DA. NMDA receptor hypofunction, parvalbumin-positive neurons, and cortical gamma oscillations in schizophrenia. Schizophrenia bulletin. 2012 9;38(5):950–7. 10.1093/schbul/sbs010 22355184 PMC3446219

[17] Weickert CS, Fung SJ, Catts VS, Schofield PR, Allen KM, Moore LT, et al Molecular evidence of N-methyl-D-aspartate receptor hypofunction in schizophrenia. Molecular psychiatry. 2012 10 16 10.1038/mp.2012.137 . 23070074 PMC3807670

