# Supplementary material for: STEP Levels Are Unchanged in Pre-Frontal Cortex and Associative Striatum in Post-Mortem Human Brain Samples from Subjects with Schizophrenia, Bipolar Disorder and Major Depressive Disorder
Source: PLoS One. 2015 Mar 18;10(3):e0121744. doi: 10.1371/journal.pone.0121744 (PMC4364624; doi:10.1371/journal.pone.0121744)
Supplement: S1 File — Normalized expression data for the Affymetrix U133_Plus2 probeset with the highest intensity for STEP are shown for PFC (top) and associative striatum (bottom). No groups had a statistically significant difference from the control group in any probeset. Fig. B. Parvalbumin mRNA is significantly reduced in PFC from schizophrenia subjects as measured by RT-PCR (**p<0.01, ***p<0.0001). Fig. C. Top: Western blot using STEP KO mouse striatal lysates to validate the polyclonal STEP antibody used to measure human STEP protein. Bottom: Western blot probing with the same STEP antibody evaluating recombinant human STEP46, full-length STEP61, and a variant of STEP61 missing a 23 amino acid proline-rich juxtamembrane domain (PRJD). Table A. STEP mRNA levels in cortex: results for testing significance of fixed effects in mixed model. Table B. STEP61 protein in cortex: initial results for testing significance of fixed effects in the full mixed model, including all interactions between covariates and the CASE factor. Table C. STEP61 protein in cortex: results of multiple comparisons tests for pairwise differences amongst covariate-adjusted means. Table D. STEP61 protein in striatum: initial results for testing significance of fixed effects in the full mixed model, including all interactions between covariates and the CASE factor. Table E. STEP61 protein in striatum: results of multiple comparisons tests for pairwise differences amongst covariate-adjusted means. Table F. STEP46 protein in striatum: initial results for testing significance of fixed effects in the full mixed model, including all interactions between covariates and CASE factor. Table G. STEP46 protein in striatum: results of multiple comparisons tests for pairwise differences amongst covariate-adjusted means. (DOCX) [file pone.0121744.s001.docx]

**Supplementary Methods:**

*Sample Processing*

Frozen samples of human Brodmann Area 46 and associative striatum were obtained from Dr. David Lewis (University of Pittsburgh). Subjects were selected to provide age- and gender-matched tetrads of schizophrenia, bipolar disorder, MDD and controls (n=19 per group). One set of samples was collected in Trizol and phenol-chloroform RNA extraction was performed. RNA was hybridized to U133_Plus2 Affymetrix whole genome microarray chips. Data were normalized by RMA, subjected to pairwise comparison followed by Benjamini and Hochberg False Discovery Rate correction (FDR).

*Statistical Analysis – RNA*

To evaluate differential mRNA expression for STEP transcripts, we employed mixed model ANCOVA model. In this model normalized RQ values served as response variable, disease group was used as fixed factor, tetrad as random factor, while age, sex, tobacco use at time of death, manner of death, PMI, RIN, and pH were used as covariates. Prior to statistical analysis, the response variable was log10 transformed, and normalized to control group for each transcript; extreme outliers outside of the 2IQR window were removed. We tested sequentially for the presence of interactions between disease group and covariates, no significant interactions were detected, and therefore the model included only linear terms for covariates. Least-squares means of disease states and control were evaluated by two types of multiple hypothesis testing adjustments: the Tukey-Kramer adjustment across disease groups independently for each combination of probe and brain region, as well as the Benjamini-Hochberg adjustment across all tests involving particular brain region. All statistical tests were performed two-tailed at a 5% level of significance. R statistical software version 3.1.0 was used for all analyses.

*Statistical Analysis - Protein*

An analysis of covariance (ANCOVA) mixed model([1](#_ENREF_1)) was used to assess significance of pairwise differences between the four cases (disease states: control, bipolar, major depressive disorder, and schizophrenia), while controlling for other potential factors that could affect measured protein levels in this assay.  The response variables were brain-enriched protein tyrosine phosphatase (STEP), sampled from the cortex using STEP61, and striatum using STEP61 and STEP46, measured by the western blot method and normalized by background correction. The initial full model included CASE as a fixed effect, SEX, AGE, and postmortem interval (PMI) as covariates, interactions between CASE and the covariates, and TETRAD and GEL as random effects, with a separate covariance structure for each random effect. Non-statistically significant covariates and interactions were removed sequentially from the model. Each combination of probe and brain region was modeled separately. Planned multiple comparisons tests for pairwise differences in covariate-adjusted least square means of the four CASE levels were performed using Tukey-Kramer and simulated multiple comparisons procedures. All statistical tests were performed two-tailed at a 5% level of significance.  SAS software version 9.3 was used for all analyses.

*Data preprocessing*

One value for STEP61 in striatum and three values STEP61 in cortex appeared to be unusual (moderately larger) than the rest of the data, but there was no a priory reason to exclude these values; all collected data were used for analysis. Model residuals for all response variables did not follow a normal distribution when assessed by a quantile-quantile (Q-Q) plot. The response variable values were transformed using a base 10 logarithm, which noticeably improved the Q-Q residual plots. Therefore, all analyses were performed using log10-transformed values. Three STEP61 values were not greater than zero (0, -0.02, -0.03) due to background corrected normalization; these three values were assigned the value of 0.01 so that the log10-transformed values were defined and used in the analysis.

*Results for STEP61 in Cortex*

The full model, which included interactions between all covariates and the CASE factor, did not show any statistically significant interaction effects (Table B), therefore all interaction terms were excluded from the model. The simplified model revealed strong association between covariates and STEP61 levels for PMI (F=10.77, p=0.0011) and AGE (F=11.24, p=0.0009) and relatively weak association with SEX (F=4.52, p=0.0341). The CASE factor was not significant, (F=1.09, p=0.3611), and multiple comparisons tests for pairwise differences among covariate-adjusted CASE means did not reveal any significant findings (Table C).

*Results for STEP61 in Striatum*

For STEP61, the full model, which included interactions between all covariates and the CASE factor, did not show any statistically significant interaction effects (Table D), therefore interaction terms were excluded from the model. The simplified model excluded the interactions, and kept the three covariates terms, two of which were statistically significant, PMI (F=20.04, p<0.0001), SEX (F=6.89, p=0.0089), but not AGE (F=0.17, p=0.6822). The CASE factor was not significant, (F=0.06, p=0.9790), and multiple comparisons tests for pairwise differences among covariate-adjusted CASE means did not reveal any significant findings (Table E). Finally, excluding the non-significant AGE covariate did not change the statistical conclusions.

*Results for STEP46 in Striatum*

For STEP46, the full model, which included interactions between all covariates and the CASE factor, did not show any statistically significant interaction effects (Table F), therefore interaction terms were excluded from the model. The next model excluded the interactions, and kept the three covariate terms, two of which were statistically significant, PMI (F=15.01, p=0.0001), SEX (F=9.35, p=0.0023), but not AGE (F=0.82, p=0.3649). The CASE factor was not significant, (F=0.46, p=0.7099), and multiple comparisons tests for pairwise differences among covariate-adjusted CASE means did not reveal any significant findings (Table G). Finally, excluding non-significant AGE covariate did not change the statistical conclusions.

**Figure A.**


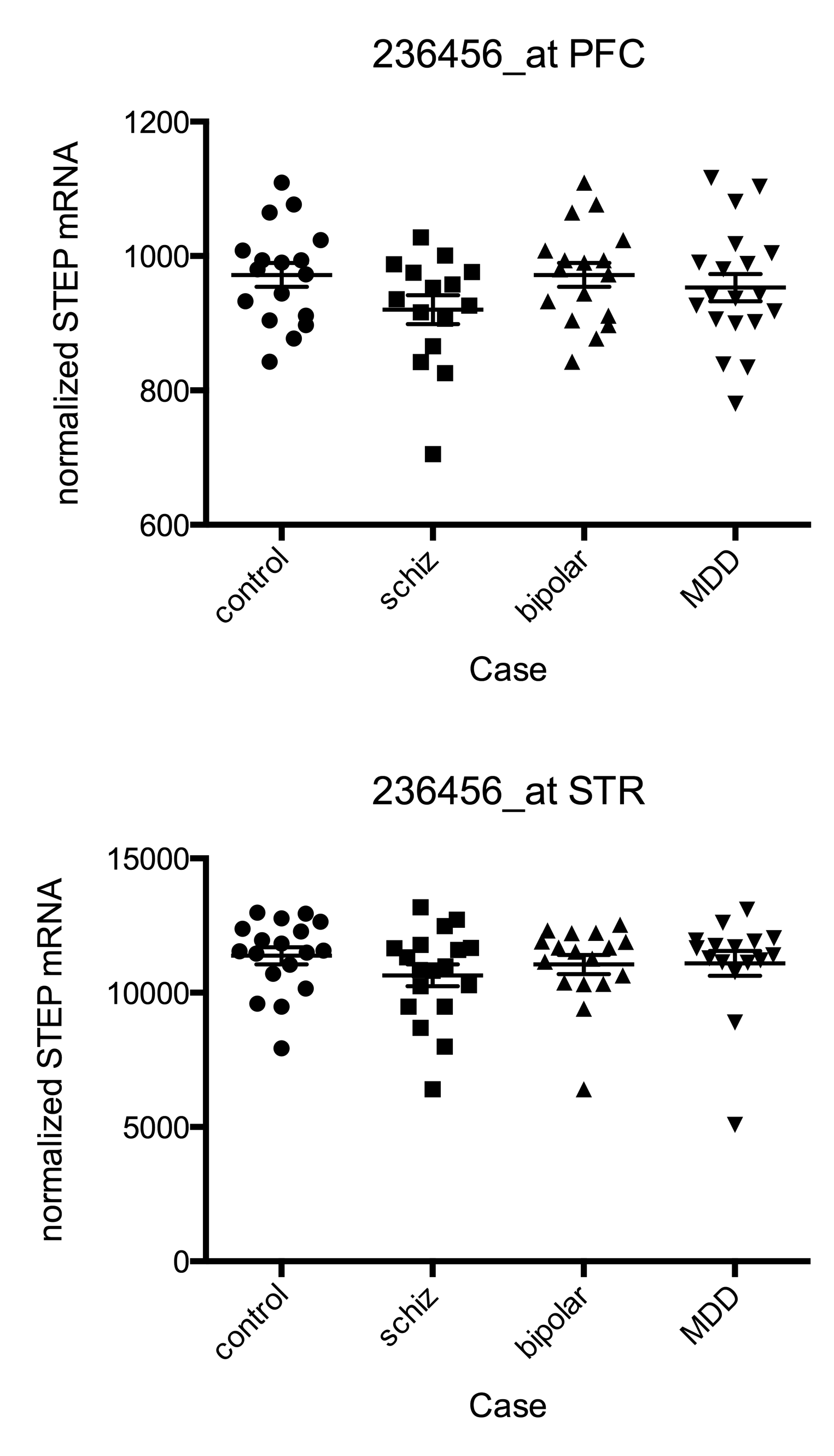


**Figure B**.


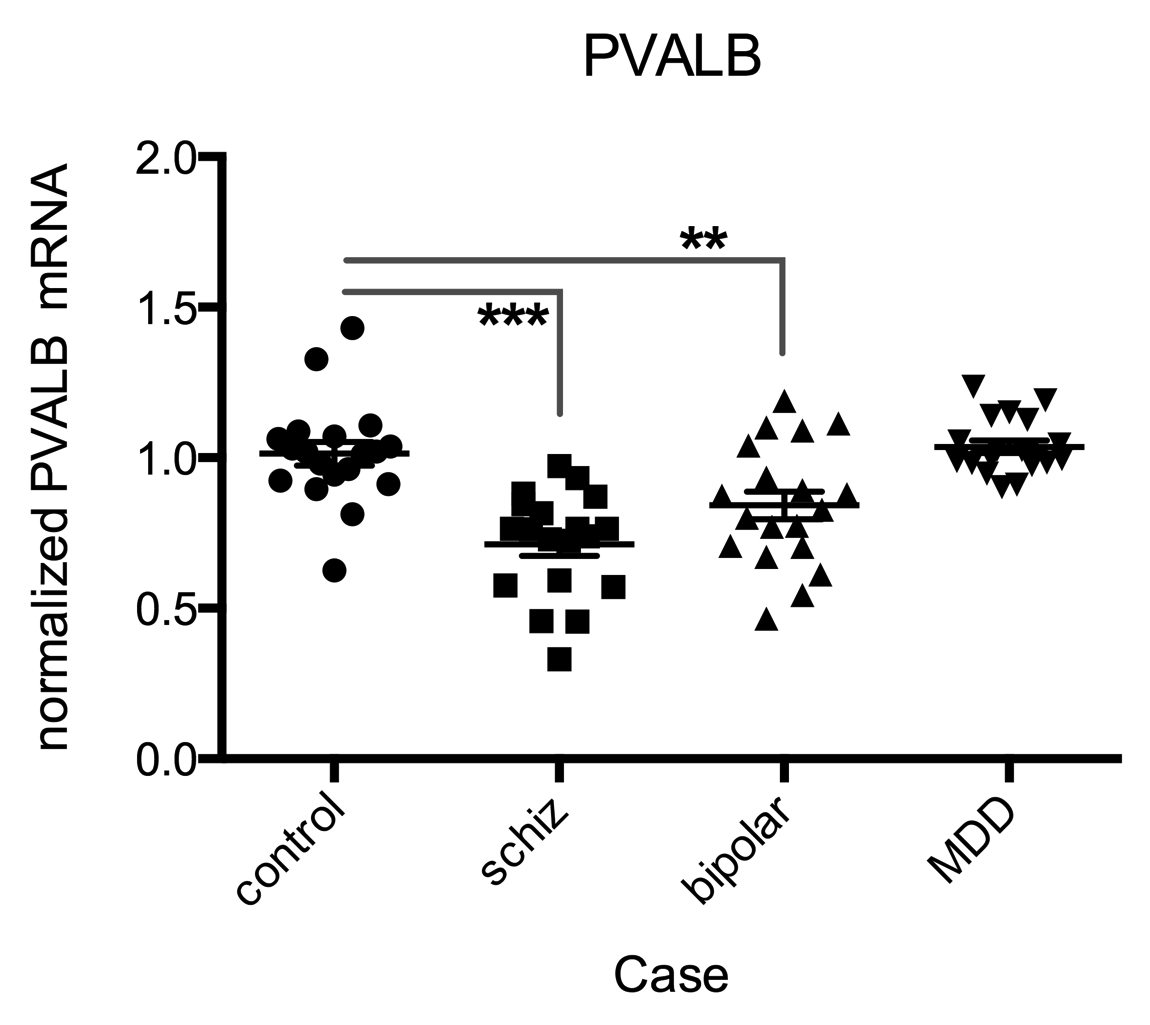


**Figure C.**


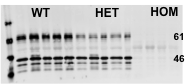


**
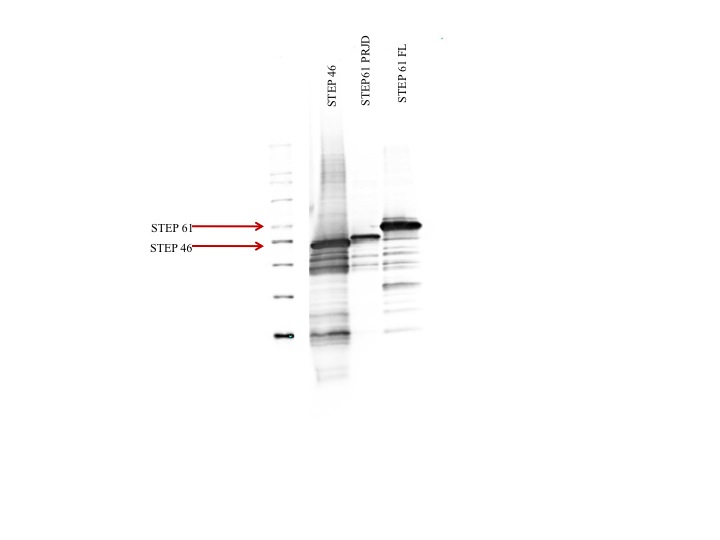
**

**Table A.**

| Assay | Sum Sq | Mean Sq | NumDF | DenDF | F.value | Pr(>F) | terms |
| --- | --- | --- | --- | --- | --- | --- | --- |
| Hs00377290_m1 | 0.034 | 0.011 | 3 | 52 | 0.1078 | 0.9551 | Subject_Group |
| Hs00377290_m1 | 0.291 | 0.291 | 1 | 29 | 2.2983 | 0.1404 | PMI |
| Hs00377290_m1 | 0.166 | 0.166 | 1 | 23 | 0.9674 | 0.3356 | Age |
| Hs00377290_m1 | 0.452 | 0.452 | 1 | 63 | 1.9961 | 0.1626 | pH |
| Hs00377290_m1 | 0.000 | 0.000 | 1 | 14 | 0.0041 | 0.9497 | Sex |
| Hs00377290_m1 | 0.374 | 0.187 | 2 | 60 | 1.0527 | 0.3553 | MOD |
| Hs00377290_m1 | 0.037 | 0.018 | 2 | 62 | 0.1028 | 0.9024 | Tob_ATOD |
| Hs00377913_m1 | 0.026 | 0.009 | 3 | 52 | 0.1929 | 0.9008 | Subject_Group |
| Hs00377913_m1 | 0.440 | 0.440 | 1 | 31 | 3.7803 | 0.0611 | PMI |
| Hs00377913_m1 | 0.541 | 0.541 | 1 | 24 | 3.4881 | 0.0741 | Age |
| Hs00377913_m1 | 0.687 | 0.687 | 1 | 62 | 4.0432 | 0.0487 | pH |
| Hs00377913_m1 | 0.021 | 0.021 | 1 | 14 | 0.1134 | 0.7415 | Sex |
| Hs00377913_m1 | 0.068 | 0.034 | 2 | 61 | 0.1720 | 0.8424 | MOD |
| Hs00377913_m1 | 0.012 | 0.006 | 2 | 61 | 0.0356 | 0.9651 | Tob_ATOD |
| Hs00986488_g1 | 0.611 | 0.204 | 3 | 51 | 0.8190 | 0.4894 | Subject_Group |
| Hs00986488_g1 | 0.076 | 0.076 | 1 | 41 | 0.7312 | 0.3975 | PMI |
| Hs00986488_g1 | 0.032 | 0.032 | 1 | 29 | 0.2050 | 0.6541 | Age |
| Hs00986488_g1 | 0.008 | 0.008 | 1 | 59 | 0.0018 | 0.9666 | pH |
| Hs00986488_g1 | 0.002 | 0.002 | 1 | 14 | 0.0000 | 0.9996 | Sex |
| Hs00986488_g1 | 0.954 | 0.477 | 2 | 61 | 1.5012 | 0.2310 | MOD |
| Hs00986488_g1 | 0.273 | 0.136 | 2 | 57 | 0.4029 | 0.6703 | Tob_ATOD |

**Table B.**

| Type 3 Tests of Fixed Effects | | | | |
| --- | --- | --- | --- | --- |
| Effect | Num DF | Den DF | F Value | Pr > F |
| CASE | 3 | 60 | 2.17 | 0.1012 |
| PMI | 1 | 456 | 11.47 | 0.0008 |
| PMI*CASE | 3 | 456 | 1.52 | 0.2074 |
| Sex | 1 | 456 | 3.92 | 0.0484 |
| CASE*Sex | 3 | 456 | 0.58 | 0.6300 |
| Age | 1 | 456 | 7.45 | 0.0066 |
| Age*CASE | 3 | 456 | 1.62 | 0.1844 |

**Table C.**

| Case 1 | Case 2 | Estimate | Standard Error | t Value | Pr > \|t\| | Tukey  Adj P | Simulate Adj P |
| --- | --- | --- | --- | --- | --- | --- | --- |
| Schizophrenia | MDD | -0.04920 | 0.04630 | -1.06 | 0.2917 | 0.7132 | 0.7182 |
| Schizophrenia | Bipolar | -0.08010 | 0.04649 | -1.72 | 0.0894 | 0.3199 | 0.3234 |
| Schizophrenia | Control | -0.02478 | 0.04660 | -0.53 | 0.5966 | 0.9510 | 0.9522 |
| MDD | Bipolar | -0.03090 | 0.04648 | -0.66 | 0.5084 | 0.9099 | 0.9118 |
| MDD | Control | 0.02442 | 0.04658 | 0.52 | 0.6018 | 0.9529 | 0.9541 |
| Bipolar | Control | 0.05532 | 0.04660 | 1.19 | 0.2392 | 0.6370 | 0.6400 |

**Table D.**

| Type 3 Tests of Fixed Effects | | | | |
| --- | --- | --- | --- | --- |
| Effect | Num DF | Den DF | F Value | Pr > F |
| CASE | 3 | 60 | 0.73 | 0.5402 |
| PMI | 1 | 540 | 17.39 | <.0001 |
| PMI*CASE | 3 | 540 | 0.29 | 0.8309 |
| Sex | 1 | 540 | 6.18 | 0.0132 |
| CASE*Sex | 3 | 540 | 0.53 | 0.6617 |
| Age | 1 | 540 | 0.09 | 0.7654 |
| Age*CASE | 3 | 540 | 0.89 | 0.4448 |

**Table E.**

| Case 1 | Case 2 | Estimate | Standard Error | t Value | Pr > \|t\| | Tukey Adj P | Simulate Adj P |
| --- | --- | --- | --- | --- | --- | --- | --- |
| Schizophrenia | MDD | -0.02138 | 0.09664 | -0.22 | 0.8250 | 0.9962 | 0.9956 |
| Schizophrenia | Bipolar | -0.02342 | 0.09687 | -0.24 | 0.8090 | 0.9950 | 0.9942 |
| Schizophrenia | Control | 0.01017 | 0.09669 | 0.11 | 0.9162 | 0.9996 | 0.9998 |
| MDD | Bipolar | -0.00204 | 0.09685 | -0.02 | 0.9832 | 1.0000 | 1.0000 |
| MDD | Control | 0.03156 | 0.09670 | 0.33 | 0.7443 | 0.9880 | 0.9875 |
| Bipolar | Control | 0.03360 | 0.09715 | 0.35 | 0.7296 | 0.9858 | 0.9856 |

**Table F.**

| Type 3 Tests of Fixed Effects | | | | |
| --- | --- | --- | --- | --- |
| Effect | Num DF | Den DF | F Value | Pr > F |
| CASE | 3 | 60 | 1.16 | 0.3309 |
| PMI | 1 | 540 | 15.59 | <.0001 |
| PMI*CASE | 3 | 540 | 0.32 | 0.8098 |
| Sex | 1 | 540 | 9.74 | 0.0019 |
| CASE*Sex | 3 | 540 | 0.2 | 0.8938 |
| Age | 1 | 540 | 0.31 | 0.5802 |
| Age*CASE | 3 | 540 | 1.84 | 0.1393 |

**Table G.**

| Case 1 | Case 2 | Estimate | Standard Error | t Value | Pr > \|t\| | Tukey Adj P | Simulate Adj P |
| --- | --- | --- | --- | --- | --- | --- | --- |
| Schizophrenia | MDD | 0.05381 | 0.1070 | 0.50 | 0.6151 | 0.9583 | 0.9581 |
| Schizophrenia | Bipolar | 0.04076 | 0.1072 | 0.38 | 0.7040 | 0.9813 | 0.9810 |
| Schizophrenia | Control | 0.11500 | 0.1070 | 1.07 | 0.2831 | 0.7053 | 0.7086 |
| MDD | Bipolar | -0.01305 | 0.1072 | -0.12 | 0.9031 | 0.9994 | 0.9993 |
| MDD | Control | 0.06119 | 0.1070 | 0.57 | 0.5678 | 0.9405 | 0.9403 |
| Bipolar | Control | 0.07424 | 0.1075 | 0.69 | 0.4903 | 0.9008 | 0.8988 |

**References**

1. Littell RC. SAS for mixed models. 2nd ed. Cary, N.C.: SAS Institute, Inc.; 2006. xii, 814 p. p.
